# Standardization of the Manufacturing Process of Bee Venom Pharmacopuncture Containing Melittin as the Active Ingredient

**DOI:** 10.1155/2018/2353280

**Published:** 2018-02-25

**Authors:** Yoonmi Lee, Sung-Geun Kim, In-Su Kim, Hwa-Dong Lee

**Affiliations:** Traditional Korean Medicine Technology Division, R&D Development, Korean Medicine Preparation Team, National Development Institute of Korean Medicine (NIKOM), 94 Hwarang-ro, Gyeongsan-si, Gyeongsangbuk-do, Republic of Korea

## Abstract

**Background:**

Pharmacopuncture is a unique treatment in oriental medicine that combines chemical stimulation with conventional acupuncture. However, there are no standardized methods for preparing the herbal medicines used in pharmacopuncture, and it is not clear whether the active ingredients are safe and stable. Several studies have investigated nonstandardized preparation processes, but few investigations have addressed safety and preparation methods. Pharmacopuncture may provide an alternative treatment for incurable diseases. However, it must be as valid and safe as standardized medicine. In this way, the present project may contribute to the industrialization of medicine in Korea. It may also expand health insurance coverage by promoting evidence-based medical insurance benefits. Thus, the present study attempted to standardize and improve the raw materials, preparation, and efficacy of bee venom pharmacopuncture (BVP), which is a highly effective technique in oriental medicine.

**Method:**

To purify the crude bee venom, the extract was subjected to a stepped-gradient open column (ODS-A; 120 Å, 150 mesh). Using this method, the yield of melittin was significantly increased and the allergen proteins were effectively removed. The melittin content of the purified bee venom was determined using HPLC, and the product was then diluted to 0.1 mg/mL using injection water in preparation for BVP.

**Results:**

In the present study, we standardized the purification process to provide safe and stable BVP by increasing the main effective components and eliminating allergens. This study will be seminal in the industrialization and regulation of BVP.

**Conclusion:**

We developed an effective strategy for melittin purification and allergen removal from bee venom to create safe BVP.

## 1. Introduction

Acupuncture has only been used for 60 years in Korean medicine. However, since the treatment was commercialized, many studies have confirmed its efficacy. Although herbal acupuncture developed from acupuncture, its mechanism of action differs somewhat. Herbal acupuncture smooths the flow of blood, which is referred to as “energy” in oriental medicine. Furthermore, the medicine contains concentrated herbal ingredients that work simultaneously, thus surpassing the efficacy of acupuncture itself. 

Until recently, there was no proper English word for herbal acupuncture. However, the term “pharmacopuncture” was registered in the 2017 medical academic information classification system (MeSH), which is used by the US National Library of Medicine (NLM) to link academic information in the healthcare field. Additionally, the term “pharmacopuncture” has been added to the new index of PubMed, which is the world's largest medical journal database.

Pharmacopuncture has strong anti-inflammatory and pain-relieving effects because it directly treats the acupuncture point. In one survey of patients who had visited oriental medicine hospitals, 48% of responders preferred pharmacopuncture to other oriental medicine treatments, because it caused a rapid decrease in pain [1, 2]. Moreover, the safety investigation suggested that acupuncture/pharmacopuncture led to a lower range, frequency, and severity of significant adverse events [3].

The venom of the European honey bee* (Apis mellifera)* comprises a mixture of proteins, peptides, and other small molecules. In bee venom pharmacopuncture (BVP), which has pain-relieving and anti-inflammatory effects, the venom is injected at appropriate doses onto acupuncture points that are selected through syndrome differentiation [4]. BVP has significant therapeutic effects on degenerative knee and rheumatoid arthritis [5–9]. The main active component of bee venom pharmacopuncture (BVP) is melittin: a peptide with antimicrobial, antitumor, and anti-inflammatory effects. In oriental medicine, honey bee venom products containing about 50% melittin are widely used for BVP. However, these products also contain the proteins phospholipase A2 (PLA2) and apamin, which are major allergens as they are capable of inducing the IgE response in susceptible individuals, according to the International Union of Immunological Societies (IUIS) [10–12]. Thus, to protect patients against side effects of BVP, both of these allergens must be effectively removed.

Previous studies have demonstrated that the purification of bee venom is a challenging task, as it requires a series of separation and purification steps [13–15]. Thus, researchers have not yet established the appropriate separation conditions for completely removing the allergen proteins while still obtaining a high yield of melittin. In the present study, we developed an effective strategy for melittin purification from bee venom. Using this method, the yield of melittin significantly increased, and the allergen proteins (apamin and PLA2) were effectively removed. The current study may help researchers to develop high quality BVP medicines. It may also expand the coverage of medical insurance by providing a basis for quality control, standardization, and good manufacturing practice (GMP) of BVP drugs.

## 2. Materials and Methods

### 2.1. Bee Venom

Crude bee venom was purchased from various manufacturers based on quality test results. The medicines were then compared with crude bee venom and with each other. Ultimately, four manufacturers were chosen: Chung-Jin Biotech, Bi-sen, and two local producers from Bong-hwa and Kyung Buk, South Korea.

### 2.2. General

High-performance liquid chromatography (HPLC) was performed with a C18-5E YMC packed column (5 *μ*m, 4.6 × 150 mm) using a Waters Alliance UV detector. Solvents for extraction, partition, thin-layer chromatography (TLC), and HPLC were distilled from HPLC grade solvents. The TLC plates used were Silica gel 60 F254 (Art. 1.05554, Merck) and RP-18 F254s (Art. 1.05560, Merck).

### 2.3. Isolation and Purification

#### 2.3.1. Solvent Stability Test

Melittin, the main active ingredient of bee venom, is a protein that is reduced or destroyed by heat, acids, bases, and so on. In the present study, ethanol was used as a solvent because it does not affect the melittin content during purification and analysis of raw bee venom. More specifically, the stability of melittin in 50% aqueous ethanol solution was investigated, and ethanol was used as a developing solvent in this experiment, because it did not change the melittin content in aqueous solution. In addition, the apamin content decreased in 50% aqueous ethanol solution.

#### 2.3.2. Isolation Scheme

Crude bee venom was isolated and purified in a g/mL dilution. This 10% diluted sample was subjected to a stepped-gradient open column (ODS-A, 120 Å, and 150 meshes) that was eluted using 0%–80% ethanol, affording 13 fractions.

#### 2.3.3. Isolation and Purity Verification

Each of the separated materials obtained through the open column, as well as their purity, were determined using HPLC. The separated components and their degree of purification were then compared with standard reagents. Apamin, PLA2, and melittin standard reagents were prepared at concentrations of 0.1 mg/mL, and their contents were confirmed. HPLC was carried out using a reversed-phase YMC C18 (5 *μ*m, 4.6 × 150 mm) that was eluted using a 10%–90% methanol-gradient menu system.

### 2.4. BVP Manufacturing

After removal of the allergen from raw bee venom and filtering of the purified melittin using membrane filters (pore size: 0.45–0.2 *μ*m), the melittin was subdivided into 2.25 mL vials.

### 2.5. BVP Quality Management

#### 2.5.1. Safety and Stability Evaluation

Changes in the melittin's composition were observed by applying the above manufacturing process and the quality control method to the prototype product using the raw materials for distribution. To confirm the stability of the BVP using various additives, we used the pH compensator that was used for preparation.

## 3. Results

### 3.1. Bee Venom

To find high quality raw material, the melittin content of different products was determined using HPLC analysis. Of the four crude bee venoms used, we found that the Bi-sen product contained the highest amount of melittin (35.75%; Table 1).

### 3.2. Isolation and Purification

#### 3.2.1. Solvent Stability

**Figure 1:**
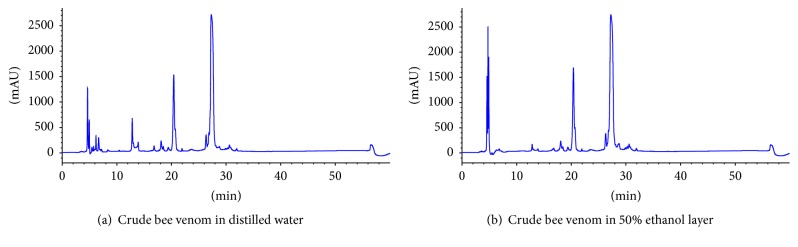
*Solvent stability*: Ethanol was considered a good solvent for reducing the side effects of BVP therapeutics. Detection wavelength: UV 220 nm column (YMC C18; 5 *μ*m, 4.6 × 150 mm), flow rate: 0.4 mL/min, sample injection amount: 30 *μ*L, mobile phase conditions: 0.1% trifluoroacetic acid in H_2_O, and 0.1% trifluoroacetic acid in acetonitrile (gradient).

Changes in melittin content were measured using ethanol, which does not affect melittin content during the analysis and purification of bee venom raw materials. HPLC confirmed that, in a 50% ethanol aqueous solution, the melittin content was stable, but the apamin content was significantly decreased (Figure 1). These results suggest that ethanol is a good solvent for reducing the side effects of BVP.

#### 3.2.2. Isolation of the Compounds

To isolate and purify the active component of crude bee venom (10 g/mL), the raw venom was partitioned into 13 fractions (Table 2). According to the corresponding HPLC profiles, Fractions 1–5 (~10% ethanol layer) contained null compounds. In Fraction 6, apamin appeared for the first time, and Fraction 7 contained both apamin and PLA2. Melittin was eluted in Fraction 10; however, it was mixed with PLA2. Pure melittin was obtained in the 70%–80% ethanol layer.

#### 3.2.3. Purity Verification

**Figure 2:**
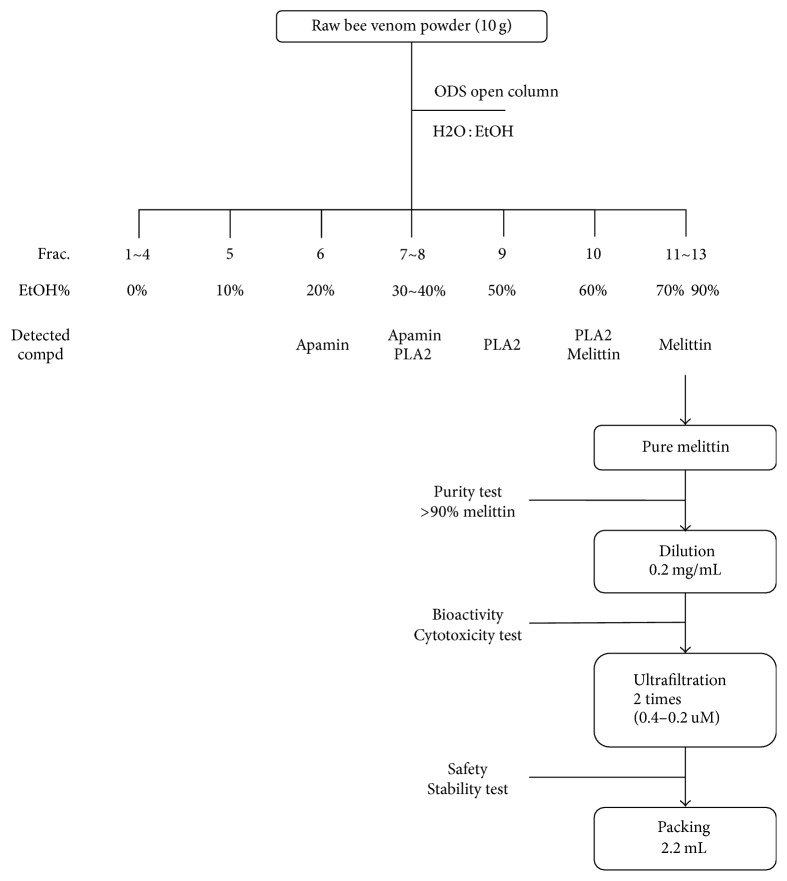
Purification process of bee venom pharmacopuncture (BVP) from raw material. The product was packaged at a good manufacturing practice (GMP) facility.

**Figure 3:**
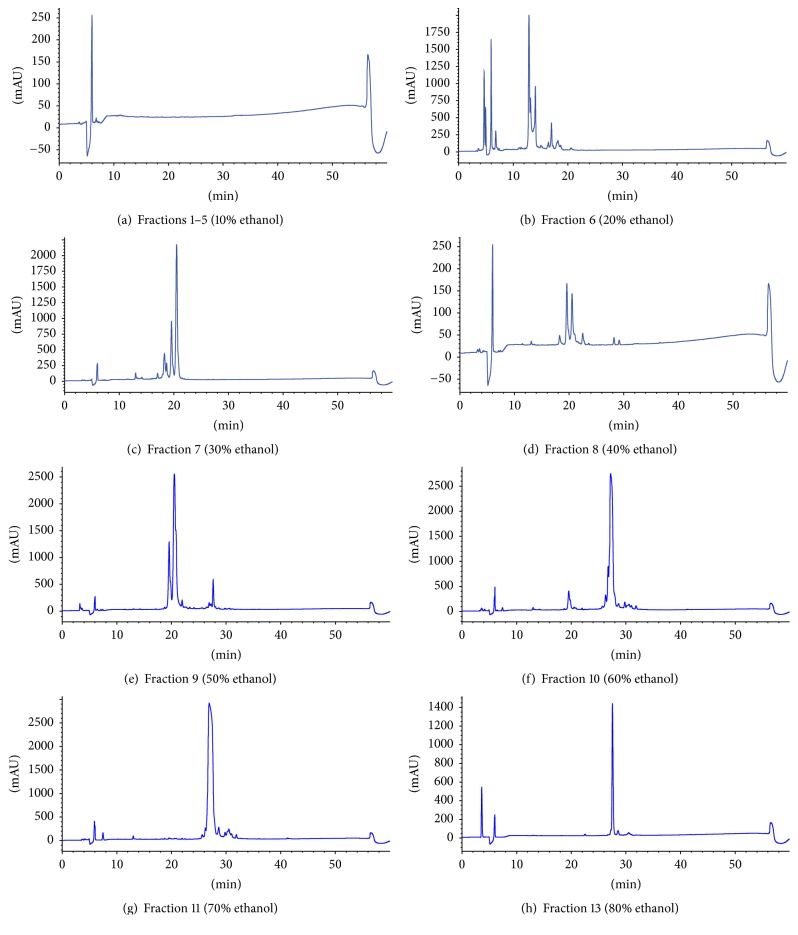
Separation of sequential compounds according to solvent polarity (apamin, PLA2, and melittin). In total, 13 fractions were isolated from the crude bee venom. Pure melittin was obtained in the 70%–80% ethanol layer.

**Figure 4:**
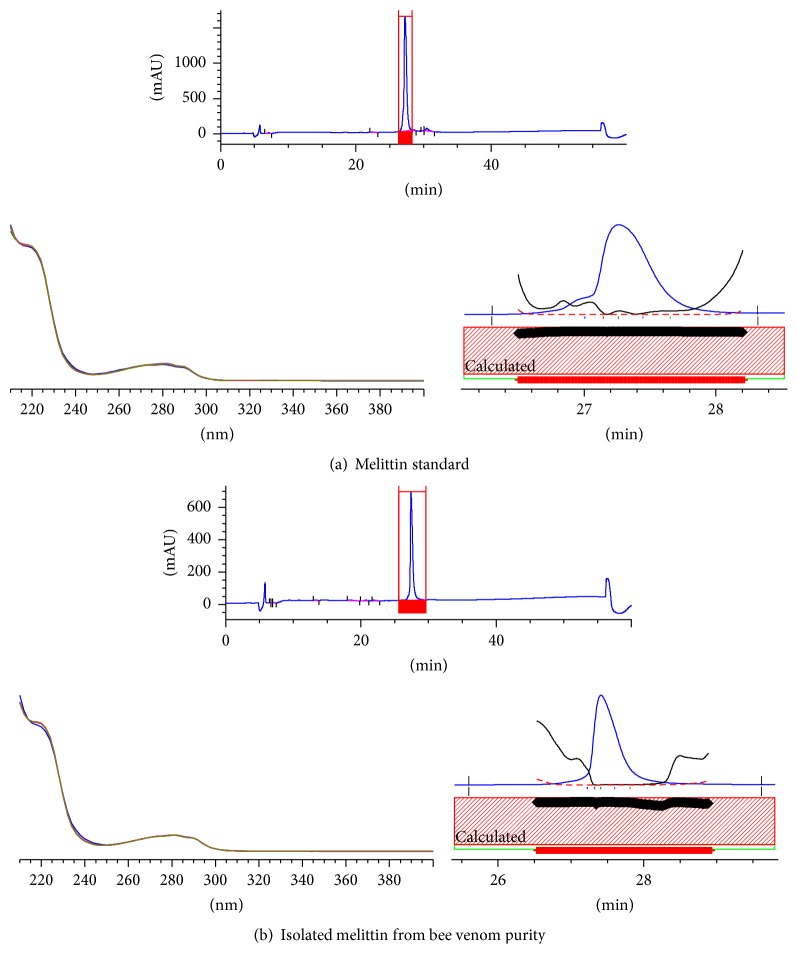
Determination of the purity of the separated melittin (a) and comparison with standard commercial melittin (b). In the comparison with the melittin standard, the purity was 99.4%, and the melittin content of purified bee venom was 99% higher than the commercial standard.

The composition of each fraction obtained through open column chromatography was determined by HPLC analysis, using apamin, PLA2, and melittin as standard compounds (Figure 2). Using the standard components, apamin was detected at 12 minutes, PLA2 in two peaks at 18 and 19 minutes, and melittin at 27 minutes. Melittin was detected from Fraction 11 (70% ethanol layer) and the peak area (%) was found to be about 98% (Figure 3, Table 3). The standard purity of the melittin was 99.4%, and melittin content of the purified bee venom was 99% higher than the commercial standard (Figure 4). The total melittin yield was 63%, and its purity was about 92%–99% after separation and purification.

### 3.3. BVP Manufacturing

The purified bee venom was concentrated and lyophilized (concentrated under reduced pressure) to produce a powder. The melittin content in the purified bee venom was determined using HPLC. The venom was then diluted to a concentration of 0.1 mg/mL, which is used in BVP, using water that had been injected through a 0.2 *μ*m membrane. Vials were filled with 2.25 mL of this drug solution. All these procedures were performed at an aseptic GMP facility (Figure 2).

### 3.4. BVP Quality Management

#### 3.4.1. Safety Evaluation

To ensure that the BVP was safe, we compared the efficacy and safety of original bee venom with those of purified bee venom that had been filtered for PLA2 and histamine, as reported previously [10].

Bee venom for BVP is produced using a medicine preparation process that ensures safety and lack of heavy metals. Thus, the evaluation items are the purity test and the heavy metal test. The purity test confirmed that the herbicide had dissolved and that there were no heavy metals (lead, cadmium, arsenic, and mercury), insoluble particulate matter, insoluble water, sterility, or endotoxins. Thus, based on these standards, the purified bee venom appeared to be appropriate (Table 4).

#### 3.4.2. Stability Evaluation

**Figure 5:**
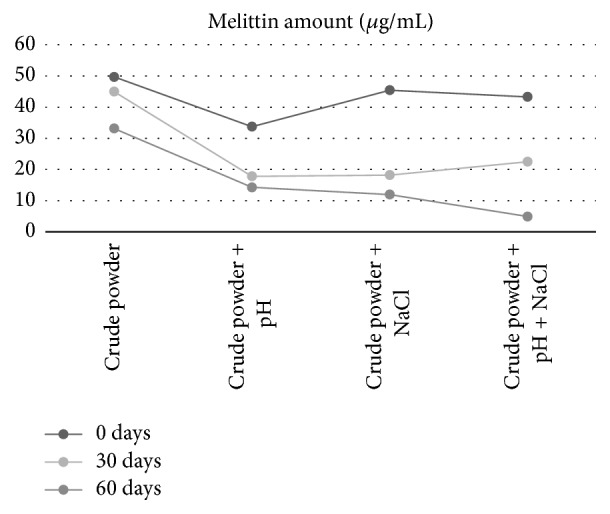
Evaluation of the stability of the bee venom with different additives. The changes of melittin components were examined for 6 months; it was found that melittin was highly stable in pH- and salinity-free pharmacopuncture.

Changes in melittin composition and purity were observed by applying the above manufacturing process and the quality control method to the prototype product using the raw materials for distribution. The pH compensator was used to confirm the stability of BVP produced using various additives (Figure 5). Changes in the melittin content were examined for 6 months, and it was found that melittin was highly stable in both pH-free and salinity-free pharmacopuncture.

## 4. Discussion

As oriental medicine develops, social interest and research into its effects are growing. In addition, unlike injections, pharmacopuncture uses acupuncture points to reduce pain and quickly identify its cause. However, because pharmacopuncture has not been standardized, regulated, and industrialized, it is not clear whether the procedure is safe and stable. The most important issue for pharmacopuncture is safety. Therefore, if pharmacopuncture is to be a pharmaceutical industry, safe medicine should be manufactured and standardized. Currently, China is actively producing and supplying medicinal herbs. To publicize this oriental medicine, China is also actively investing in the medicine business. Chinese pharmacopuncture often uses two or more kinds of medicines from a single medicinal herb or material; these are administered to patients in various formulations.

Therefore, to ensure the safety and the stability of this treatment, it is urgent that researchers standardize pharmacopuncture. In this way, the therapy could be popularized through the pharmaceutical industry.

To our knowledge, the present study was the first that aimed to standardize and improve the raw materials, preparation, and efficacy of BVP, which is a highly effective oriental medicinal treatment. Crude bee venom (Bi-sen) was isolated and purified in a 1 g/mL dilution. In total, 13 fractions were isolated from the crude bee venom. Pure melittin was obtained in the 70%–80% ethanol layer. In comparison with the melittin standard, its purity was 99.4%, and melittin content of our purified bee venom was 99% higher than the commercial standard. Our total melittin yield was 63% and its purity was 92%–99% after separation and purification. The content of melittin in our purified bee venom was determined by HPLC; the melittin was then diluted to 0.1 mg/mL in preparation for BVP. All these procedures were performed at an aseptic GMP facility.

## 5. Conclusions

This experiment aimed to separate melittin from crude bee venom to produce safe, effective, and high-concentration standardized medicines for pharmacopuncture. We standardized the manufacturing process to provide safe and stable BVP by increasing the concentrations of the effective components and eliminating allergens. Thus, this study will be seminal in the industrialization and regulation of BVP.

## Figures and Tables

**Table 1:** Comparing raw bee venom by production area.

Division	Apamin (%)	PLA2 (%)	Melittin (%)
Chung-Jin	37.240	12.631	32.245
Bi-sen	44.019	14.016	35.751
Local 1	37.959	10.771	34.432
Local 2	13.772	1.935	1.292

*Content Standards*. Standard product apamin, PLA2, and melittin (0.1 mg content).

**Table 2:** Separate substances (detected compound) according to their solvent formulations.

Fr.	Solvent gradient	Amount	Detected compound
	(H_2_O : ethanol)	(mL)	
1	0	50 (each)	ND
2	0		ND
3	0		ND
4	0		ND
5	10%		ND
6	20%		Apamin
7	30%		Apamin, PLA2
8	40%		Apamin, PLA2
9	50%		PLA2
10	60%		PLA2, melittin

11	70%	100	Melittin

12	70%	50	Melittin

13	80%	100	Melittin

ND: not detected.

**Table 3:** Component content eluted by fractions.

Change in component contents						
Fraction number	6	7	8	9	10	11
Apamin (%)	42.32	2.04	2.19	0.35	0.4	0.62
PLA2 (%)	0.59	20.17	24.64	6.16	1.59	0.16
PLA2 (%)	1.66	77.79	67.93	88.15	8.75	0.33
Melittin (%)	-	-	5.25	5.35	89.26	98.89

**Table 4:** Safety evaluation of bee venom pharmacopuncture.

Bee venom pharmacopuncture	
Purity test	
Dissolution state	ND
Lead	0 ppm
Cadmium	0.0 ppm
Arsenic	0 ppm
Mercury	0.0 ppm
Insoluble particulate matter	ND
Soluble particulate matter	ND
Sterility test^1^	ND
Endotoxin test^2^	ND

^1^Sterility test: direct method using liquid thioglycolic acid medium and soybean casein digestion medium. ^2^Endotoxin test: using Pierce® LAL Chromogenic Endotoxin Quantitation Kit (determination coefficient [*R*2] ≥ 0.9932).

## References

[1] Korean Pharmacopuncture Institute *Korean Pharmacopuncture Institute Compilation: A Pharmacopuncture Prepared from Herbs And Clinical Application*.

[2] Korean Pharmacopuncture Institute (2012). Chapter 1, Definition and history. *Pharmacopuncturology*.

[3] Kim M.-R., Shin J.-S., Lee J. (2016). Safety of acupuncture and pharmacopuncture in 80,523 musculoskeletal disorder patients: A retrospective review of internal safety inspection and electronic medical records. *Medicine (United States)*.

[4] Ahn Y.-J., Shin J.-S., Lee J. (2016). Safety of essential bee venom pharmacopuncture as assessed in a randomized controlled double-blind trial. *Journal of Ethnopharmacology*.

[5] Ko H. K. (1992). Experimental Studies on the Effect of Bee Venom Theraphy on the Analgesic, Antipyretic and Anti-inflammatory Action. *Korean journal of oriental medicine*.

[6] Kwon K. R., Koh H. K., Kim Y. S., Park Y. B., Kim C. H., Kang S. K. (1997). The Effect of Bee Venom Aqua-acupuncture on the Antitumor and Immune Response in the Epithelioma by 3-MCA. *Journal of Korean Acupuncture Moxibustion Society*.

[7] Lim J.-A., Sung-Nam K., Sung-Young L. (2005). The clinical study on bee venom acupuncture treatment on osteoarthritis of knee joint. *Journal of Pharmacopuncture*.

[8] Wu-Hao W., Kyu-Beom A., Jin-Kang L., Hyoung-Seok J. (2001). Clinical investigation compared with the effects of the bee-venom acupuncture on knee joint with osteoarthritis. *Journal of Pharmacopuncture*.

[9] Lee K. M., Lee K. S., Yem S. C. (2004). A Clinical study of Bee-venom acupuncture treatment on protrusion disc Patients. *Journal of Korean Acupuncture Moxibustion Society*.

[10] Moreno M., Giralt E. (2015). Three valuable peptides from bee and wasp venoms for therapeutic and biotechnological use: Melittin, apamin and mastoparan. *Toxins*.

[11] Spoerri E., Jentsch J., Glees P. (1975). Apamin from bee venom. Effects of the neurotoxin on subcellular particles of neural cultures. *FEBS Letters*.

[12] Freeman C. M., Catlow C. R. A., Hemmings A. M., Hider R. C. (1986). The conformation of apamin. *FEBS Letters*.

[13] Maulet Y., Brodbeck U., Fulpius B. W. (1982). Purification from bee venom of melittin devoid of phospholipase A2 contamination. *Analytical Biochemistry*.

[14] Zhu W., Wang B., Zhu X. (2002). Isolation and purification of BV I -2H from bee venom and analysis of its biological action. *Chinese Science Bulletin*.

[15] Moon D.-O., Park S.-Y., Lee K.-J. (2007). Bee venom and melittin reduce proinflammatory mediators in lipopolysaccharide-stimulated BV2 microglia. *International Immunopharmacology*.

